# Plastic responses to competition: Does bacteriocin production increase in the presence of nonself competitors?

**DOI:** 10.1002/ece3.4203

**Published:** 2018-06-25

**Authors:** Amrita Bhattacharya, Hannah Tae‐Young Pak, Farrah Bashey

**Affiliations:** ^1^ Department of Biology Indiana University Bloomington Indiana

**Keywords:** adaptation, adaptive phenotypic plasticity, allelopathy, bacteriocins, competition, competition‐sensing hypothesis

## Abstract

Anticompetitor traits such as the production of allelopathic toxins can confer significant competitive benefits but are often costly to produce. Evolution of these traits may be facilitated by environment‐specific induction; however, the extent to which costly anticompetitor traits are induced by competitors is not well explored. Here, we addressed this question using bacteriocins, which are highly specific, proteinaceous anticompetitor toxins, produced by most lineages of bacteria and archaea. We tested the prediction that bacteriocin production is phenotypically plastic and induced by the presence of competitors by examining bacteriocin production in the presence and absence of nonself competitors over the course of growth of a producing strain. Our results show that bacteriocin production is detectable only at high cell densities, when competition for resources is high. However, the amount of bacteriocin activity was not significantly different in the presence vs. the absence of nonself competitors. These results suggest that bacteriocin production is either (a) canalized, constitutively produced by a fixed frequency of cells in the population or (b) induced by generic cues of competition, rather than specific self/nonself discrimination. Such a nonspecific response to competition could be favored in the natural environment where competition is ubiquitous.

## 1 INTRODUCTION

Competition is one of the most pervasive biological interactions, and it plays a key role in structuring ecological communities (Tilman, 1982). Anticompetitor strategies such as the production of allelopathic chemicals can confer a competitive advantage to the producers, but such chemicals are often associated with high costs (Lankau, 2008; Pintar & Starmer, 2003; Riley & Wertz, 2002b). How are such costly competitive strategies maintained? Phenotypic plasticity may serve as one important mechanism for the maintenance of costly traits as the costs may be mitigated through conditional, environment‐specific expression of the traits. For example, predator‐induced morphological changes allow protection in the presence of a predator, but also allow for reduced costs in the absence of predation (Baur, Binder, & Benz, 1991; Laforsch & Tollrian, 2004; Lively, 1986b). Similarly, costly chemical defenses are well documented to be induced by herbivory (Dicke & Hilker, 2003; Haukioja, 1991; Zangerl, 2003). However, the degree to which equally costly anticompetitor chemicals are induced by competition has not been well explored. To date, just a few studies have found evidence for competition‐induced increases in the production of allelopathic compounds in some plants, algae, and bacteria (Korgaonkar & Whiteley, 2011; Lankau & Kliebenstein, 2009; Rasher & Hay, 2014).

Despite these examples of adaptive plasticity, not all costly traits will be phenotypically plastic. Phenotypically plastic genotypes need to maintain sensory and regulatory machinery required for detecting and responding to relevant changes in the environment, and may therefore incur costs relative to nonplastic genotypes (reviewed in DeWitt, Sih, & Wilson, 1998; Murren et al., 2015). Other factors such as the timeliness of phenotypic response following environmental change, as well as reliability of environmental cues, may impose limitations on the benefits gained from plasticity (Lively, 1986a; Moran, 1992; Padilla & Adolph, 1996; Scheiner & Holt, 2012). Further, environmental heterogeneity over relevant timescales for the trait in question is a key criterion for the evolution of adaptive plasticity (Bradshaw, 1965; Moran, 1992). Thus, whether a costly competitive trait is phenotypically plastic and induced by the presence of a competitor is likely to be determined by the interplay of multiple ecological and physiological parameters.

Bacteriocins provide an excellent opportunity to investigate whether costly anticompetitor chemicals are competitor induced. Bacteriocins are a class of allelopathic compounds produced by bacteria that can confer a competitive advantage by targeting and killing closely related competitors. A vast majority of archaea and almost every known lineage of bacteria are known to produce at least one bacteriocin (James, Lazdunski, & Patty, 1991; Klaenhammer, 1988; Riley & Wertz, 2002a). However, bacteriocin production is highly costly, requiring the lysis of producing cells in some species, and the costs of production are often greater than the direct benefits gained through competitor killing (Riley & Chavan, 2006; Riley & Wertz, 2002b; Wloch‐Salamon, Gerla, Hoekstra, & de Visser, 2008). As such, bacteriocin production is considered one of the clearest examples of spite in nature, whereby the producers incur a substantial cost and reduce the fitness of targeted competitor cells (Gardner, West, & Buckling, 2004; West, Griffin, Gardner, & Diggle, 2006). Theory predicts that resource allocation to bacteriocin production should be increased when the producing lineage occurs at intermediate frequencies with a competing lineage (Gardner et al., 2004). Further, empirical work has shown that the relative fitness of a bacteriocin‐producing lineage is highest when the starting frequencies of the producer and sensitive strains are 50:50 (Inglis, Gardner, Cornelis, & Buckling, 2009). Therefore, it is reasonable to hypothesize that bacteriocin production is phenotypically plastic, and increases in more competitive environments. This hypothesis remains largely untested.

The ability to sense appropriate changes in the environment is critical for phenotypic plasticity to be adaptive. Bacteria are known to respond to changes in their social environments (Kümmerli, Jiricny, Clarke, West, & Griffin, 2009; Rumbaugh et al., 2009) and communicate with each other through diverse mechanisms including quorum sensing (Lerat & Moran, 2004; Waters & Bassler, 2005) and contact‐dependent interactions (Basler, Ho, & Mekalanos, 2013; Gibbs, Urbanowski, & Greenberg, 2008; Ruhe, Low, & Hayes, 2013). Particularly relevant to bacteriocin production is the recently proposed “competition‐sensing hypothesis”, which posits that bacteria have evolved the ability to sense and respond to competition (Cornforth & Foster, 2013). A meta‐analysis of bacteriocin regulatory pathways revealed that production is frequently regulated by stress response pathways that are triggered by competition‐related stressors such as nutrient stress and cellular damage. Conversely, bacteriocin production is rarely associated with stress response pathways that are sensitive to stressors like heat or osmotic stress, which are not competition‐related (Cornforth & Foster, 2013).

**Figure 1:**
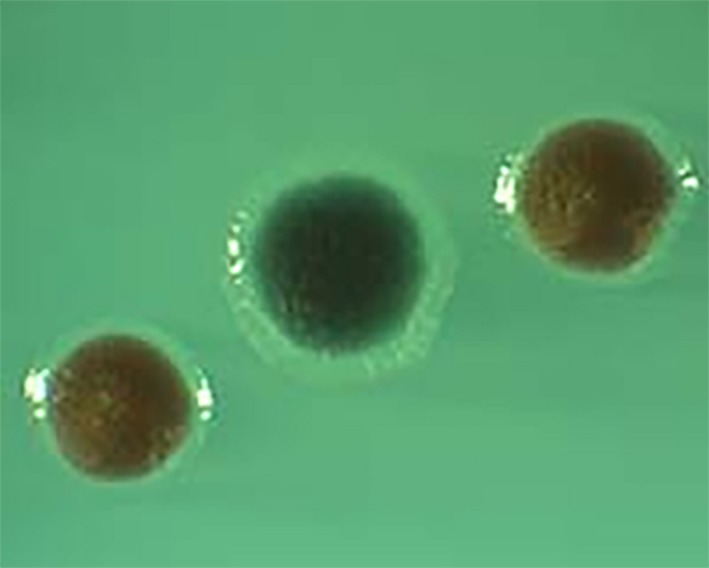
Image showing colonies of natural *Xenorhabdus* isolates used in the study. The producer strain *Xenorhabdus koppenhoeferi* isolate 46 (Kop 46), grows as small, maroon colonies, while colonies of the competitor strain *Xenorhabdus bovienii* isolate 59 (Bov 59) are larger and blue

Here, we test whether bacteriocin production plastically increases in more competitive environments by examining bacteriocin production at different stages of growth in the presence and absence of nonself competitors. We test this prediction using natural isolates of *Xenorhabdus spp*. (Figure 1), which have been a key study system to investigate the maintenance of bacteriocin production in nature (Hawlena, Bashey, & Lively, 2010; Hawlena, Bashey, & Lively, 2012; Hawlena, Bashey, Mendes‐Soares, & Lively, 2010). *Xenorhabdus spp*. are insect‐parasitic bacteria that gain entry into an insect host by forming symbioses with entomopathogenic nematodes (Herbert & Goodrich‐Blair, 2007; Martens, Heungens, & Goodrich‐Blair, 2003; Stock & Blair, 2008). Within the insect host, bacteriocin‐mediated interactions occur and are known to affect the outcome of competition (Bashey, Hawlena, & Lively, 2013; Bashey, Young, Hawlena, & Lively, 2012). We use field‐collected isolates of *Xenorhabdus spp*., which have a rich, competitive environment in nature where they may frequently, but not always, encounter nonself competitors within the insect host.

## 2 MATERIALS AND METHODS

### 2.1 Experimental design

**Figure 2:**
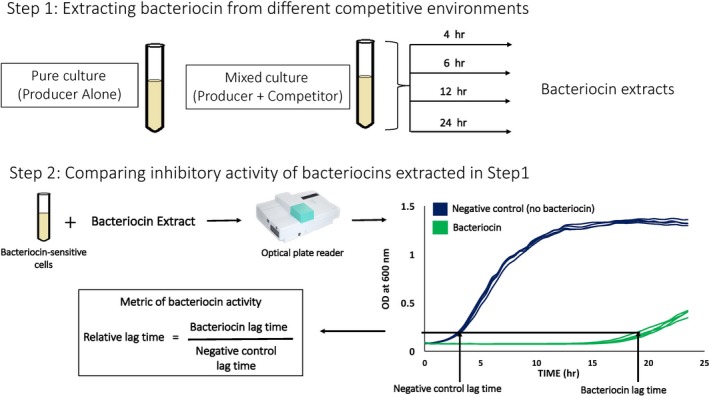
Schematic showing the experimental design and methods. The competitive environment of a bacteriocin‐producing lineage was manipulated by either growing the producer strain alone in the pure culture or in the presence of a competing strain in mixed culture. Bacteriocin extracts were collected from these treatments at four different time points (4, 6, 12, and 24 hr). These extracts were subsequently tested for inhibitory activity using a growth‐inhibition assay. A fixed amount of a bacteriocin extract was added to a starting culture of bacteriocin‐sensitive cells. The growth of these cultures was tracked in an optical plate reader which measured OD_600_ readings at 30‐min intervals for 24 hr. An extract derived from a pure culture of the competitor cells which contains no bacteriocin was used as a negative control for the bacteriocin extracts. Data from the optical plate reader yield growth curves as shown. Four curves for each treatment represent curves from four replicate wells on the optical plate reader. Lag times were estimated for all wells, and the average of four wells for each treatment was used to calculate relative lag time as shown

A producer strain was grown alone (pure culture) or in the presence of a nonself competitor strain (mixed culture) and bacteriocin production was examined at multiple time points over the course of growth (Figure 2). Producer cell densities were measured at each time point examined. To measure bacteriocin production, filter‐sterilized supernatants from the cultures were used in growth‐inhibition assays, where the growth trajectory of a sensitive strain was used as a bioassay of the amount of bacteriocin in the supernatant. Specifically, the delay in growth of the sensitive strain induced by the presence of a bacteriocin extract was used to derive a metric of bacteriocin activity (Figure 2). As a negative control, cell‐free supernatant from cultures of the competitor strain grown alone was run alongside the two producer treatments and was processed similarly at each time point during the bacteriocin production phase of the experiment, as well as during the growth‐inhibition assays. (Figure 2).

### 2.2 Bacterial strains

The bacteriocin‐producing clone used in this study was a natural isolate of *Xenorhabdus koppenhoeferi* (Kop46) which is known to produce a bacteriocin that can suppress the growth of a sympatrically isolated clone of *Xenorhabdus bovienii* (Bov59) (Bashey et al., 2013). The competitor strain used in the experiment, Bov59R, was derived in the lab from Bov59. This strain was chosen because bacteriocin that is exposed to Bov59R cells maintains its inhibitory activity (Supporting information Figure S1). This was key for the current experiment to ensure that the inhibitory activity of the bacteriocin released by producing cells in the mixed treatment would not be subsequently altered by the presence of competitor cells. The natural isolate Bov59, which is sensitive to bacteriocin, was used as the detector strain in the growth‐inhibition bioassays. These strains of *X. koppenhoeferi* and *X*. *bovienii* are morphologically distinct (Figure 1), whereby Kop46 colonies appear maroon and Bov59/59R colonies appear blue on NBTA plates (nutrient agar supplemented with 0.0025% (w⁄ v) bromothymol blue and 0.004% (w⁄ v) triphenyltetrazolium chloride, pH = 8). In addition, Bov59/59R has a higher natural resistance to ampicillin than Kop46, thereby enabling further distinction on NBTA plates with 75 μg/ml ampicillin. All cultures used in the experiment were derived from freezer stocks maintained at −80°C and streaked onto NBTA plates prior to each replicate.

### 2.3 Bacteriocin production treatments

To initiate each replicate, individual colonies of each strain were picked from freezer streaks and inoculated into 5 ml LB media (Difco) in 20‐ml culture tubes. Cultures were grown overnight at 28°C and then used to inoculate fresh LB broth to establish the three experimental groups. The mixed culture treatment was established by adding a fixed volume (50 μl) each of the producer Kop46 and the competitor Bov59R overnight cultures into 5 ml LB. The producer alone treatment was established by adding 50 μl of a Kop46 overnight culture and 50 μl of LB to 5 ml LB. Similarly, the negative control treatment was established by adding 50 μl of the competitor Bov59R overnight culture and 50 μl LB into 5 ml LB. All culture tubes were incubated with shaking (120 rpm) at 28°C.

Bacteriocin extracts were collected at four time points over the course of growth: 4 hr (early‐exponenetial), 6 hr (mid‐exponential), 12 hr (late‐exponential), and 24 hr (stationary) phases. Cultures tubes were destructively sampled at each time point. A total of 10 producer and competitor colonies were examined at 24 hr and six colonies each were tested at the earlier time points. A small aliquot (100 μl) was taken from each culture tube both immediately after setup and just before bacteriocin collection to calculate initial and final cell densities and to ensure the absence of contamination. These aliquots were diluted and plated on NBTA agar. Plates were incubated at 28°C for 48 hr before colonies were counted. Cell density estimates confirmed that initial densities of producer and competitor cells in the mixed treatment were within the same order of magnitude (~10^6^ c.f.u/ml), and the average frequency of producer cells was 0.61± 0.04 (mean ± 1 *SEM*).

Bacteriocin extracts were collected by centrifuging cultures at 1,620 *g* for 5 min, and the supernatant was filtered through 0.45 μm filters (Acrodisc). This procedure allows the proteinaceous bacteriocin to pass through while eliminating any cells in the extract. These bacteriocin extracts were stored at 4°C until use in the growth‐inhibition assays. Growth‐inhibition assays were performed within 7 days of bacteriocin extraction.

### 2.4 Growth‐inhibition assay

The growth‐inhibition assay was used to measure and compare the degree of growth inhibition induced by the application of bacteriocin extracts to a starting culture of bacteriocin‐sensitive cells. Each bacteriocin extract was examined on four replicate wells in a 100‐well plate reader (Honeycomb plate, Growth Curves USA). Each well contained a 200 μl solution of a bacteriocin extract and starting cell culture in 1:5 ratio by volume. Varying cell densities of starting cell cultures were used (10^6^, 10^5^, or 10^4^ c.f.u/ml) for bacteriocin extracts from different time points. The difference between the lag times induced by the negative control and bacteriocin samples increases with decreasing starting cell densities of the detector strain, facilitating the detection of bacteriocin‐mediated inhibition (Supporting information Figure S2). Consequently, lower cell densities were used for the earlier time points where bacteriocin concentrations were low to facilitate detection of bacteriocin.

Every plate had internal control wells with un‐inoculated media to rule out contamination. Culture growth in the wells was measured by a Bioscreen plate reader (Growth Curves USA) in which the plates were incubated at 28°C with continuous shaking at medium amplitude. O.D._600_ was recorded every 30 min for 24 hr.

### 2.5 Follow‐up growth rate analysis

To test whether the presence of Bov59R imposes competition on the producer cells, the growth rate of producer cells growing in the presence and absence of the competitor was estimated in an additional experiment. Paired replicates of the producer alone and mixed treatments were established using 10 different producer colonies, and cell densities were estimated at 0, 6, and 24 hr from each tube. 100 μl aliquots were used to make serial dilutions that were plated on NBTA plates to estimate cell densities. Growth rate of the producer over 6 and 24 hr was estimated for each tube as ln[producer density at final time point/producer density at 0 hr].

### 2.6 Statistical analyses

The optical density measures for each well were used to compute lag times using the software program GrowthRates 2.1 (Hall, Acar, Nandipati, & Barlow, 2013). Lag time represents a measure of the delay observed before a culture moves into the exponential growth phase. GrowthRates 2.1 calculates lag time as the time point at which the extrapolated slope of the exponential phase line (lnOD vs. time) meets the *x*‐axis. Every well was manually examined for model accuracy in estimating lag time.

The average lag time value of the four wells containing the same bacteriocin extract was used to calculate the relative lag time for each extract (Figure 2). The relative lag time induced by a given bacteriocin extract was calculated as the ratio of the average lag time value induced by the extract to the average lag time value induced by the corresponding negative control extract (extracts from competitor strain Bov59R pure cultures, Figure 2). A relative lag time value of 1 indicates no deviation from the negative control, and values significantly greater than 1 indicate inhibitory activity.

Relative lag times for bacteriocin extracted from pure cultures (Kop46 alone) and mixed cultures (Kop46 + Bov59R) were compared at each of the four time points. A mixed‐model analysis of variance was conducted in SAS 9.4 with bacteriocin source (pure or mixed culture) as fixed effect and “colony identity” and “experimental run” as random effects. Each experimental run was performed using one or two colonies of the producer and competitor.

Colony counts were used to compare the initial and final cell densities of the bacteriocin producer cells and to calculate producer growth rate (ln[producer density at final time point/producer density at 0 hr]) across treatments. Mixed‐model analyses of variance were used with “treatment” (pure or mixed culture) as fixed effect and “colony identity” and “experimental run” as random effects.

For the follow‐up growth rate analysis experiment, producer growth rates in the presence and absence of Bov59R were compared using mixed‐model analyses of variance with “treatment” (pure or mixed culture) as fixed effect and “colony identity” as random effect.

## 3 RESULTS

**Figure 3:**
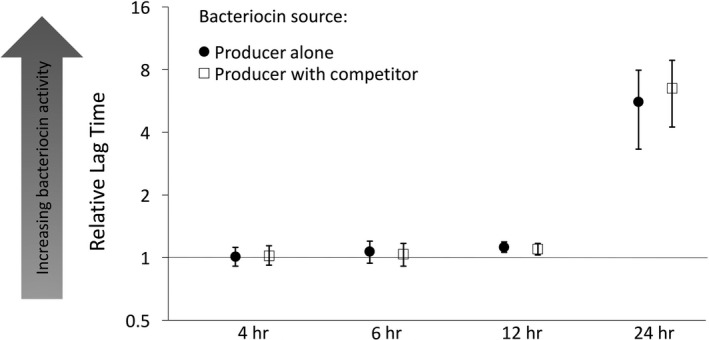
Relative lag times (±95% confidence intervals) induced by bacteriocin extracts collected from cultures of the producer growing alone (filled‐in circles) and producer growing in mixed cultures with equal starting frequencies of a competing strain (empty squares). The *x*‐axis represents the time points at which bacteriocin extracts were collected over the course of growth. Each time point was destructively sampled. Relative lag time values greater than 1 indicate significant inhibitory activity. Relative lag time values are calculated as shown in Figure 2

Bacteriocin extracts were collected from the pure and mixed cultures at four distinct time points (4, 6, 12, and 24 hr). Their inhibitory activities were measured by examining the lag time they induced in the growth of the sensitive strain. Significant bacteriocin‐mediated inhibitory activity was detected only at 12 and 24 hr (Figure 3, 95% C.I. do not overlap with 1). No bacteriocin‐mediated inhibition was detectable at the earlier time points (Figure 3, 95% C.I. overlap with 1). There was no significant difference between the relative lag times induced by the extracts derived from pure cultures and mixed cultures at any of the four time points examined (Figure 3, 4 hr: *F*_1,10_ = 0.06, *p* = 0.81; 6 hr: *F*_1,10_ = 0.20, *p* = 0.66; 12 hr: *F*_1,10_ = 0.29, *p* = 0.60, and 24 hr: *F*_1,18_ = 0.40, *p* = 0.53).

**Figure 4:**
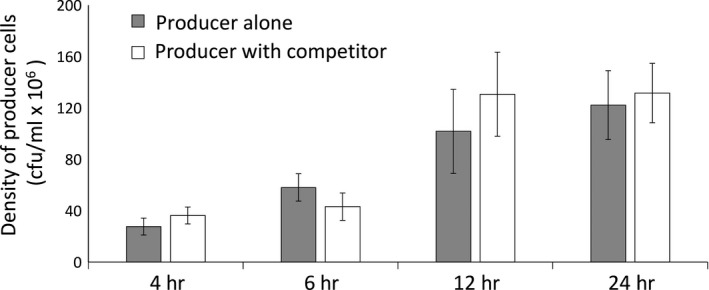
The density (± 1 *SEM*) of cells from the bacteriocin producer strain in the pure and mixed cultures at the time of bacteriocin extractions

**Figure 5:**
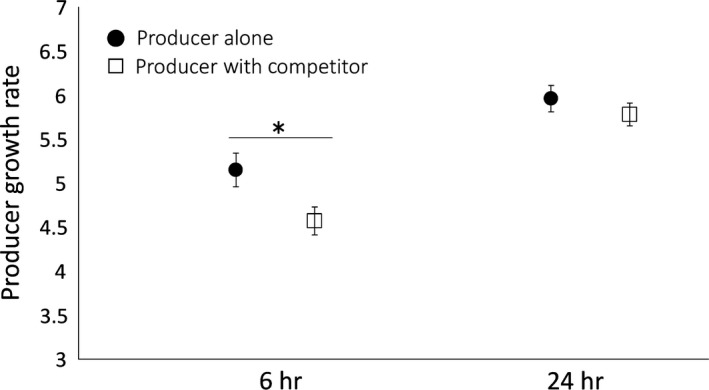
The growth rate of the producer strain (±1 *SEM*) in the pure and mixed cultures measured at 6 and 24 hr. Growth rate at each time point was calculated as ln[final density of producer cells/initial density of producer cells]. The * indicates a significant difference between treatments (*p* < 0.05)

We examined producer cell densities at the time of bacteriocin extraction to establish that bacteriocin activity was not confounded by differences in the total number of producer cells. Comparisons of producer cell densities between the pure and mixed cultures were not significantly different at any time point (Figure 4, 4 hr: *F*_1,10_ = 0.84, *p* = 0.32; 6 hr: *F*_1,10_ = 1.28, *p* = 0.28; 12 hr: *F*_1,10_ = 0.39, *p* = 0.54, and 24 hr: *F*_1,18_ = 0.30, *p* = 0.59). The density of competitor cells was reduced to, or below, our detection limit of 10^5^ c.f.u/ml at 4 hr, and 10^6^ c.f.u/ml at 6, 12, and 24 hr. Comparable producer densities between treatments indicate that on a per cell basis, the producer cells did not differ in their bacteriocin production in response to the presence of a nonself competitor. Despite the lack of significant differences in producer cell densities, comparing the growth rate of the producer strain in the pure and mixed cultures revealed an 11.6% reduction in producer growth in the mixed treatment at 6 hr; although, this difference was not significant (*F*_1,5_ = 1.20, *p* = 0.32). To determine whether this pattern was indicative of a difference in the competitive environment experienced by the producer cells, we ran 10 additional replicates of the producer alone and in the presence of Bov59R. Growth rates were found to be significantly lower in the mixed treatment at 6 hr (Figure 5, *F*_1,5_ = 10.95, *p* = 0.021). Producer growth rate was reduced by 11.3% in the follow‐up experiment, consistent with the reduction observed in the initial experiment. The difference in growth rate did not persist to 24 hr (Figure 5, *F*_1,5_ = 1.10, *p* = 0.34).

## 4 DISCUSSION

Bacteriocins are a class of allelopathic toxins that are ubiquitously produced across microbial lineages despite being highly costly (Riley & Chavan, 2006; Riley & Wertz, 2002a; Wloch‐Salamon et al., 2008). While theory predicts that allocation to bacteriocin production should be increased when producer cells occur at intermediate frequencies relative to nonself competitor cells (Gardner et al., 2004), the degree to which bacteriocin production is phenotypically plastic and induced by the presence of nonself competitors remains underexplored. We tested the plasticity hypothesis using natural isolates of *Xenorhabdus spp.,* by examining bacteriocin production at various time points over the course of growth when producer cells were grown in the pure culture, or in coculture with a nonself competitor strain. Our results show that bacteriocin production can be detected during the late exponential phase (12 hr) and the stationary phase (24 hr), regardless of the presence of nonself competitors. Thus, there is no evidence for increased bacteriocin production in the presence and absence of nonself competitors (Figure 3). While bacteriocin activity in both treatments reflected an almost sevenfold increase between 12 and 24 hr, this increase occurred without any concomitant increases in producer cell densities at these time points (Figure 4), indicating that bacteriocin production may be increasing on a per cell basis between these time points. However, bacteriocin activity detected at each time point in these experiments is cumulative and reflects the activity of bacteriocin produced over the entire course of growth until time of sampling. Therefore, the increase in bacteriocin production between 12 and 24 hr may be also explained solely by the accumulation of bacteriocin without invoking a per capita increase in toxin production. Overall, these results provide no evidence to suggest that bacteriocin production is increased in response to the presence of nonself competitors.

We set out to test the prediction that bacteriocin production increases in coculture with nonself competitors, when the benefits of toxin production can be realized by the producer population. However, bacteriocin production may not be expected to increase in the presence of nonself cells if the nonself cells do not impose increased competition on the producers. To determine whether the nonself cells used in our experiment imposed increased competition on producer cells, we compared the growth rate of the producer strain in the presence and absence of the nonself competitors at 6 and 24 hr. Results revealed that producer cells had significantly reduced growth rates in the mixed culture at 6 hr (Figure 5), indicating that the producer cells experienced increased competition due to the presence of Bov59R cells in the mixed culture early on in the experiment. Bacteriocin production was examined over the course of growth (Figure 3) encompassing time points when the presence of Bov59R imposes increased competition on producer cells in coculture. However, our results did not provide any evidence for increased bacteriocin activity in the presence of the nonself competitor at any examined time points (Figure 3).

Bacteriocin production is costly. In addition to costs of protein synthesis and genetic maintenance, many species of gram‐negative bacteria require producer cells to lyse for bacteriocin release (Riley & Chavan, 2006; Riley & Wertz, 2002b; Wloch‐Salamon et al., 2008). Consequently, only a small proportion of the producer population is actively engaged in bacteriocin production and release at a given time. In *Escherichia coli*, this frequency has been estimated to range between 0.5% and 9% using GFP reporter strains linked to bacteriocin promoters (Bayramoglu et al., 2017; Mulec et al., 2003). While similar estimates for the frequency of cells actively producing bacteriocin remain to be determined for the natural isolates of *Xenorhabdus spp.,* our functional assays clearly show no evidence in support of the hypothesis that bacteriocin production increases in the presence of nonself competitors. Recently published work demonstrates that *E. coli* populations increase colicin production in response to attacking competitors (Mavridou, Gonzalez, Kim, West, & Foster, 2018). Intriguingly, the competitor strain used in our experiment does not attack the producer strain. This contrast suggests the possibility that plastic increases in toxin production may be more likely to occur when producer cells experience direct attack, and this hypothesis may be explicitly tested in future investigations.

The time course of bacteriocin production in our results suggests that bacteriocin production increases in stationary phase. These results are consistent with previous findings using *E. coli* (Eraso, Chidambaram, & Weinstock, 1996). Taken together, these results suggest that toxin production may be triggered by generic cues associated with competition such as nutrient limitation, arising from increasing cell densities, instead of specific mechanisms of self/nonself detection. Such a mechanism of competition‐sensing via generic cues is also consistent with the “competition‐sensing hypothesis” (Cornforth & Foster, 2013). The ability of bacterial cells to detect and respond to nonself genotypes has been documented in multiple species (Basler et al., 2013; Garbeva, Silby, Raaijmakers, Levy, & de Boer, 2011; Gibbs et al., 2008; Korgaonkar & Whiteley, 2011; Unterweger, Kitaoka, Miyata, & Bachmann, 2012; Wenren et al., 2013). However, tailoring specific responses to different nonself genotypes may be highly costly and require specialized receptors to detect specific competitor cues. Moreover, competitor cues may also evolve to evade detection. Thus, it has been suggested that sensing general cues associated with competition may be more useful than relying on self/nonself recognition (Cornforth & Foster, 2013).

An important criterion for a phenotypically plastic response to be effective is its timeliness (Padilla & Adolph, 1996). Plasticity in bacteriocin production may be unfavorable if induced bacteriocin production is too slow, thereby allowing a growth advantage to the competing strain while the producer first detects and then responds by producing bacteriocins. The *Xenorhabdus spp*. isolates used in this study are entomopathogenic bacteria which form mutualistic associations with *Steinernema* nematodes to enter an insect host (Martens et al., 2003; Stock & Blair, 2008). Quick growth is crucial for the successful colonization of the insect host as well as for the competitive success of these bacteria (Bashey, Reynolds, Sarin, & Young, 2011). In fact, in some cases, a sensitive but faster growing isolate can outcompete a producer strain (Bashey et al., 2013) demonstrating the importance of timing in the within‐host competitive environment.

Further, if producers frequently encounter sensitive, nonself competitors in their natural habitat, selection for plasticity in response to a nonself competitor may not be strong. The benefits of bacteriocin production during frequent encounters with sensitive competitors may outweigh the costs of toxin production on rare occasions when the producer does not encounter a competitor. These conditions seem plausible for the species used in this study. Numerous genotypes of *Xenorhabdus spp*. have been found at an ecologically relevant scale (Hawlena, Bashey, Lively, 2010; Hawlena, Bashey, Mendes‐Soares, et al., 2010) suggesting that the presence of nonself competitors may be ubiquitous in nature, thus further reducing the selection for plasticity. Future investigations may employ experimental evolution to investigate whether bacteriocin production differentially evolves when competitors are reliably encountered in the environment or not.

In highly diverse communities with numerous producing, sensitive, and resistance genotypes, bacteriocin production may be maintained through nontransitive interactions resulting in frequency‐dependent selection (Kerr, Riley, Feldman, & Bohannan, 2002). Additionally, the prediction that bacteriocin production should be plastically increased in response to nonself competitors may not hold up if bacteriocins serve other functions in addition to anticompetitor roles. It has been suggested that for microbes that form symbioses with other organisms, antimicrobial toxins may also serve as honest signals to facilitate mutualist pairing (Hillman & Goodrich‐Blair, 2016). If bacteriocins serve as important signals to attract nematode mutualists, then production would be beneficial and necessary even in the absence of bacterial competitors. If so, once producer populations reach high densities, it may be time to find mutualist partners within the host and release bacteriocins irrespective of the presence of nonself competitors. Further investigation to examine the consequences of manipulating bacteriocin production on effective symbiont association could influence the way costs and benefits of bacteriocin production are understood, and shed light on the regulation and maintenance of bacteriocins in nature. In addition to bacteriocins, allelopathic traits in other taxa may also not be plastically responsive to the presence of nonself competitors for analogous ecological reasons. Overall, these results strongly highlight the need for more studies in the future aimed at investigating the constitutive versus inducible nature of anticompetitor traits and testing alternative predictions to develop a better understanding of how costly anticompetitor traits are maintained in nature.

## AUTHOR CONTRIBUTIONS

AB conceived and codesigned the study, conducted experiments and data analysis, and wrote the manuscript. HP conducted experiments and collected data with AB. FB codesigned the study, helped with data analysis, and writing the manuscript.

## CONFLICT OF INTEREST

None declared

## DATA ACCESSIBILITY

Data available from the Dryad Digital Repository: https://doi.org/10.5061/dryad.n0j5b19
