# Supplementary material for: Plastic responses to competition: Does bacteriocin production increase in the presence of nonself competitors?
Source: Ecol Evol. 2018 Jun 25;8(14):6880–8. doi: 10.1002/ece3.4203 (PMC6065276; doi:10.1002/ece3.4203)
Supplement: Supplementary file 1 [file ECE3-8-6880-s001.docx]

**Supplemental Materials**

**S1: Choosing the competitor strain for mixed treatment –** This assay was performed to determine whether the inhibitory activity of bacteriocin produced by the producer strain could be subsequently affected by the presence of the competitor strain in the mixed culture. To test whether exposure to cells can affect the inhibitory activity of bacteriocin, a fixed amount of chemically induced bacteriocin was either “Exposed” by applying it to a starting culture of cells (Bov59 and Bov59R) or “Unexposed” by adding it to the same volume of un-inoculated growth medium (Luria Broth). A pure culture of Bov59R with no added bacteriocin was used as a “No Bacteriocin” negative control. All four treatments were incubated at 28^o^C with shaking for 24 hours. Following incubation, supernatants were collected and a growth inhibition assay was performed to compare the bacteriocin-mediated inhibitory activity across the four groups. Here, inhibitory activity is measured as the absolute lagtime (minutes) induced by the various extracts on the growth of a starting culture of bacteriocin sensitive Bov59 cells. Absolute lag times induced by the no bacteriocin negative control, unexposed bacteriocin and the bacteriocin extracts exposed to either Bov59 or Bov59R were compared using a mixed model analysis of variance with ‘bacteriocin source’ as fixed effect and experimental replicate as random effect.


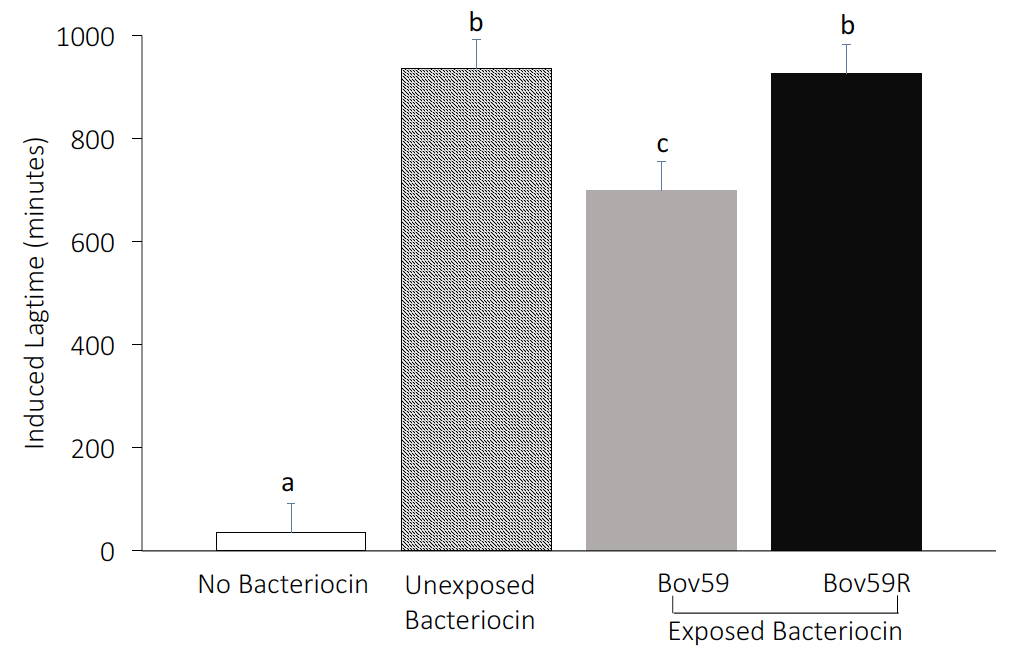


**Figure S1: Inhibitory activity of bacteriocin exposed to Bov59 and Bov59R cells in comparison with bacteriocin that is unexposed to any bacterial cells.** Bacteriocin that was previously exposed to Bov59 cells showed a significant reduction in inhibitory activity compared to bacteriocin which was previously unexposed to cells but otherwise similarly treated. In contrast, exposure to the lab-derived Bov59R strain showed the same inhibitory activity of bacteriocin as unexposed bacteriocin. Following these results the Bov59R strain was chosen as the competitor strain for the mixed treatment. Mean lagtime (+/- Standard error ) are plotted in minutes; the letters (a,b,c) indicate statistically significant differences between treatments at p<0.05.

**S2: Sensitivity of the growth inhibition assay –** The growth inhibition assay was performed using three different starting cell densities (10^^6^, 10^^5^ and 10^^4^ c.f.u/ml) of the sensitive detector culture. Three different dilutions of chemically induced bacteriocin were applied to starting cultures at each density. A no bacteriocin negative control was applied to each starting cell density as well. The difference in lagtimes induced by a bacteriocin dilution and the respective negative control was used as a metric of assay sensitivity. A greater difference in the induced lagtimes between negative control and bacteriocin samples provides an increased window for detecting bacteriocin activity. Each bacteriocin dilution- x- cell density combination was tested on four replicate wells in the optical plate reader. The induced lagtime for each well was subtracted from the mean induced lagtime of the relevant no bacteriocin control. The average differences in induced lagtimes with respective standard error of means are plotted in Figure S2.


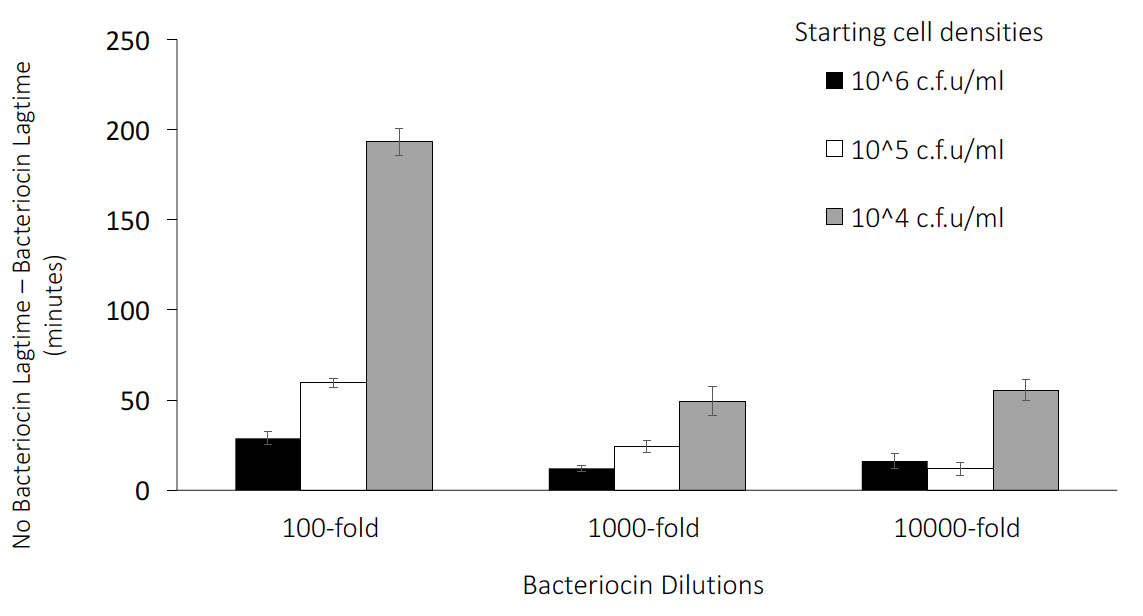


**Figure S2: Sensitivity of growth inhibition assay increases with decreasing starting cell densities of detector culture.** The difference between lagtime induced by a no bacteriocin negative control and the lagtime induced by a fixed concentration of chemically induced bacteriocin increases when growth inhibition assay is performed using lower starting cell culture densities. Shown here are three different dilutions of chemically induced bacteriocin (100-fold, 1000-fold and 10000-fold) tested across three different starting cell densities. For any bacteriocin dilution, the difference in lagtimes between negative control and bacteriocin dilution is highest at the lowest starting cell dilution. This increased difference in the lagtimes makes it more likely to detect inhibitory effects of low concentrations of bacteriocins. The error bars represent standard error of mean.
